# Cardiovascular autonomic dysfunction and oxidative stress induced by fructose overload in an experimental model of hypertension and menopause

**DOI:** 10.1186/1471-2261-14-185

**Published:** 2014-12-11

**Authors:** Filipe Fernandes Conti, Janaina de Oliveira Brito, Nathalia Bernardes, Danielle da Silva Dias, Iris Callado Sanches, Christiane Malfitano, Susana Francisca Llesuy, Maria-Claudia Irigoyen, Kátia De Angelis

**Affiliations:** Laboratory of Translational Physiology, Universidade Nove de Julho (UNINOVE), São Paulo, SP Brasil; Hypertension Unit, Heart Institute (InCor), School of Medicine, University of Sao Paulo, São Paulo, SP Brasil; Departamento de Química Analítica y Fisicoquímica, Facultad de Farmacia y Bioquímica, Universidad de Buenos Aires, Buenos Aires, Argentina

**Keywords:** Menopause, Metabolic syndrome, Heart rate variability, Blood pressure variability, Inflammation, Oxidative stress

## Abstract

**Background:**

Metabolic syndrome is characterized by the association of 3 or more risk factors, including: abdominal obesity associated with an excess of abdominal fat, insulin resistance, type 2 diabetes, dyslipidemia and hypertension. Moreover, the prevalence of hypertension and metabolic dysfunctions sharply increases after the menopause. However, the mechanisms involved in these changes are not well understood. Thus, the aim of this study was to assess the effects of fructose overload on cardiovascular autonomic modulation, inflammation and cardiac oxidative stress in an experimental model of hypertension and menopause.

**Methods:**

Female SHR rats were divided into (n = 8/group): hypertensive (H), hypertensive ovariectomized (HO) and hypertensive ovariectomized undergoing fructose overload (100 g/L in drinking water) (FHO). Arterial pressure (AP) signals were directly recorded. Cardiac autonomic modulation was evaluated by spectral analysis. Oxidative stress was evaluated in cardiac tissue.

**Results:**

AP was higher in the FHO group when compared to the other groups. Fructose overload promoted an increase in body and fat weight, triglyceride concentration and a reduction in insulin sensitivity. IL-10 was reduced in the FHO group when compared to the H group. TNF-α was higher in the FHO when compared to all other groups. Lipoperoxidation was higher and glutathione redox balance was reduced in the FHO group when compared to other groups, an indication of increased oxidative stress. A negative correlation was found between IL-10 and adipose tissue.

**Conclusion:**

Fructose overload promoted an impairment in cardiac autonomic modulation associated with inflammation and oxidative stress in hypertensive rats undergoing ovarian hormone deprivation.

## Background

Metabolic syndrome is characterized by the association of 3 or more risk factors, including: abdominal obesity associated with an excess of abdominal fat, insulin resistance, type 2 diabetes, dyslipidemia and hypertension [1]. Changes in dietary habits are among the leading contributors to the growing worldwide prevalence of metabolic syndrome, obesity, and type 2 diabetes. These changes are characterized by increased intake of simple sugars, particularly fructose, largely used in the food industry and sugar-sweetened drinks [2]. Indeed, several studies have pointed to the adverse cardiac and metabolic effects of a high fructose diet in animal models. Fructose-fed rats and mice show moderate hypertension and glucose intolerance, associated with increased levels of plasma insulin, cholesterol and triglycerides [3–5]. Our group has demonstrated that only eight weeks consumption of fructose was sufficient to induce promote significant metabolic and cardiovascular changes associated with autonomic dysfunctions in mice and rats [3, 5, 6]. Recently, we have demonstrated that increased sympathetic modulation of vessels and heart preceded the development of metabolic dysfunction in fructose fed mice [4].

However, it should be stressed that the prevalence of hypertension and metabolic dysfunctions sharply increases after menopause. The association between autonomic dysfunction and cardiovascular diseases support a link between alterations in the sympathovagal control and increased cardiovascular risk after menopause [7].

Moreover, there seems to be an association between oxidative stress and inflammation, and both would thus play a role in the development of several cardiovascular diseases [8]. However, the role of the oxidative stress and inflammation on cardio-metabolic dysfunction after ovarian hormone deprivation is yet not well understood. Thus, our study was designed to evaluate the hypothesis that in this context, fructose overload may further affect cardiovascular autonomic modulation, along with inflammation and oxidative stress in hypertensive rats undergoing ovarian hormone deprivation. In this study, we assessed the effects of fructose overload on cardiovascular autonomic modulation, inflammation and oxidative stress in an experimental model of hypertension and menopause.

## Methods

21 day old female SHRs were obtained from the Animals Facilities of the Institute of Cardiology of Rio Grande do Sul. The rats were randomly divided into hypertensive(H; n = 8), hypertensive OVX (HO; n = 8) and hypertensive OVX undergoing fructose overload (FHO; n = 8). All surgical procedures and protocols were approved by the ethics committee of Universidade Nove de Julho (Protocol AN002/12) and were conducted in accordance with the Guide for the Care and Use of Laboratory Animals, issued by the National Institutes of Health.

### Fructose overload

FHO rats received D-fructose (100 g/L) in drinking water for 19 weeks. H and HO animals received standard laboratory chow and water ad libitum. The consumption of chow and water (with or without fructose) was measured weekly.

### Ovariectomy

After 10 weeks of fructose overload, the animals were anesthetized (80 mg/kg ketamine and 12 mg/kg xylazine), and the oviduct was sectioned and the ovary removed as described in detail elsewhere [9–11]. Data from our laboratory have demonstrated that estrogen concentration, measured through immunoassay, was 39 ± 7 pg/mL in healthy female rats. However, in the present study, estrogen concentration was not detectable in the ovariectomized studied groups, thus confirming ovarian hormone deprivation [10].

### Metabolic evaluations

At the end of protocol (19 weeks of fructose overload),blood glucose and triglyceride concentrations were measured (Accucheck and Accutrend, Roche) after 4-hour fasting [4–6, 12]. For insulin tolerance test, the animals fasted for 2 hours and were then anesthetized with sodium thiopental (40 mg/Kg body weight, IP) [6, 13]. A drop of blood was collected from the tail for blood glucose level measurement at baseline and 4, 8, 12, and 16 minutes after insulin injection (0.75 U/kg). The constant rate for blood glucose disappearance (KITT) was calculated using the 0.693/t1.2 formula. Blood glucose t1/2 was calculated from the slope of the least squares analysis of blood glucose concentrations during the linear phase of decline [6, 13]. Chow and water (with or without fructose) consumption were measured weekly. The total caloric intake was calculated using 2.89 kcal per gram of chow consumed, and each ingested gram of fructose corresponded to 4.0 kcal [6].

### Cardiovascular measurements

After metabolic evaluations, rats were anesthetized with ketamine (80 mg/kg) and xylazine (12 mg/kg), and two polyethylene-tipped Tygon cannulas filled with heparinized saline were implanted into the carotid artery and jugular vein for direct measurements of AP and drug administration, respectively. During the experiment, rats received food and water ad libitum; the rats remained conscious while in their cages and were allowed to move freely during the hemodynamic experiments. The arterial cannula was connected to a transducer (Blood Pressure XDCR, Kent Scientific), and blood pressure signals were recorded for a 30-minute period using a microcomputer equipped with an analog-to-digital converter (CODAS, 2Kz, DATAQ Instruments). The recorded data were analyzed on a beat-to-beat basis to quantify changes in systolic (SAP), diastolic (DAP), and mean arterial pressure (MAP) and heart rate (HR) [5, 9, 10].

### Autonomic measurements

Time-domain analysis consisted of calculating mean pulse interval (PI) and SAP, with PI variability and SAP variability measured from its respective time series (three time series of 5 min for each animal). For frequency domain analysis, the same time series of PI and SAP were cubic splineY interpolated (250 Hz) and cubic splineY decimated so that they would be equally spaced in time after linear trend removal; power spectral density was obtained through the fast Fourier transformation. Spectral power for low-frequency (LF; 0.20-0.75 Hz) and high-frequency (HF; 0.75-4.0 Hz) bands were calculated by power spectrum density integration within each frequency bandwidth, using a customized routine (MATLAB 6.0, Mathworks) [14].

### Inflammatory markers on cardiac tissue

One day after hemodynamic evaluations the animals were killed by decapitation and the heart (ventricles) was immediately removed, rinsed in saline, and trimmed to remove fat tissue and visible connective tissue. IL-6, IL-10 and TNF-α levels were determined using a commercially available ELISA kit (R&D Systems Inc.), in accordance with the manufacturer's instructions. ELISA was performed in 96-well polystyrene microplates with a specific monoclonal antibody coating. The threshold of sensitivity for the TNF-α , IL-10 and Il-6 assays was 15.0 pg/mL. Absorbance was measured at 540 nm in a microplate reader.

### Oxidative stress profile on cardiac tissue

The cardiac tissue (~0.5 g) was cut into small pieces, placed in ice-cold buffer, and homogenized in an ultra-Turrax blender with 1 g tissue per 5 mL 150 mMKCl and 20 nM sodium phosphate buffer, pH 7.4. The homogenate was centrifuged at 600 g for 10 min at -26C.

#### Thiobarbituric acid reactive substances (TBARS)

For the TBARS assay, trichloroacetic acid (10%, w/v) was added to the homogenate to precipitate proteins and to acidify the samples [15]. This mixture was then centrifuged (10000 g, 3 min), the protein-free sample was extracted, and thiobarbituric acid (0.67%, w/v) was added to the reaction medium. The tubes were placed in a water bath (100°C) for 15 min. The absorbencies were measured at 535 nm using a spectrophotometer. The results are expressed as µmol per milligram of protein.

#### Chemiluminescence

Cardiac tissue membrane lipoperoxidation was evaluated by chemiluminescence. The chemiluminescence assay was carried out with an LKB Rack Beta liquid scintillation spectrometer 1215 (LKB Producer AB, USA) in the out-of-coincidence mode at room temperature (25°C to 27°C). The supernatants were diluted in 140 mM KCl and 20 mM sodium phosphate buffer, pH 7.4, and added to glass tubes, which were placed in scintillation vials; 3 mM tert-butylhydroperoxide was added, and chemiluminescence was determined up to the maximal level of emission [9, 16].

#### Antioxidant enzyme activities

The quantification of SOD activity, expressed as U/mg protein, was based on the inhibition of the reaction between O2˙ - and pyrogallol [17]. CAT activity was determined by measuring the decrease in H_2_O_2_ absorbance at 240 nm. CAT acitivity was expressed as μmol H_2_O_2_ reduced/min/mg protein [18]. GPx activity was expressed as nmol peroxide/hydroperoxide reduced/min/mg protein and was based on the consumption of NADPH at 480 nm [19].

#### Glutathione and GSSG concentration

To determine GSSG and total glutathione concentration, tissue was homogenized in 2 M perchloric acid and centrifuged at 1000 *g* for 10 min,and 2 M KOH was added to the supernatant. The reaction medium contained 100 mM phosphate buffer (pH 7.2), 2 mM NADPH, 0.2 U/mL glutathione reductase and 70 μM 5,5’-dithiobis (2-nitrobenzoic acid). To determine the GSSG concentration, the supernatant was neutralized with 2 M KOH and inhibited by the addition of 5 μM N-ethylmaleimide. Absorbance was read at 420 nm [20]. GSH values were determined from the total and GSSG concentration.

### Statistical analysis

Data are expressed as mean ± SEM. The Levene Test was used to evaluate data homogeneity. A one-way analysis of variance followed by the Student-Newman-Keuls test was used to compare groups. Pearson correlation analysis was used to identify an association between variables. Significance level was established at P ≤ 0.05.

## Results

Body weight and adipose tissue weight were higher in FHO group when compared to other groups. Chow consumption was similar among groups (H: 16.8 ± 2.2; HO: 11.5 ± 2.9; FHO: 14.0 ± 0.5 g/day). The FHO group, however, presented a statistically significant greater water consumption (82.9 ± 1.4 mL/day) when compared to H group (25.2 ± 0.2 mL/day) and HO group (24.6 ± 3.0 mL/day). The total caloric intake (chow + fructose) was higher in FHO when compared to other groups (FHO: 73.7 ± 0.8 vs. H: 48.7 ± 6.0; HO: 33.3 ± 8.5 kcal/day). Glycemia was similar between groups. Triglycerides levels were increased in the FHO group when compared to all other groups. Insulin sensitive was lower in FHO group when compared to the H group (Table 1).

**Table 1:** **Metabolic evaluations in studied groups**

	H	HO	HFO
**Body weight (g)**	197 ± 2	264 ± 3^†^	261 ± 2^†^
**Adipose tissue (g)**	1.91 ± 0.16	2.96 ± 0.45	5.25 ± 0.39^†¥^
**Glucose (mg/dL)**	83 ± 5.0±	84 ± 2.0	92 ± 2.1
**Triglyceride (mg/dL)**	132 ± 4.0	125 ± 6.4	160 ± 8.0^†¥^
**KITT (%/min)**	4.15 ± 0.26	4.69 ± 0.33	3.4 ± 0.28^†^

Data are reported as mean ± SEM. ^†^P < 0.05 vs. H; ^¥^P < 0.05 vs HO. KITT: constant rate for blood glucose disappearance.

The FOH group presented resting tachycardia when compared to the other groups. DAP and MAP were higher in HO group when compared to the H group and the FHO group had an additional increase in SAP, DAP and MAP (Table 2).

**Table 2:** **Hemodynamic and autonomic evaluation in studied groups**

	H	HO	FHO
**SAP (mmHg)**	174 ± 5	183 ± 6	206 ± 4^†¥^
**DAP (mmHg)**	121 ± 7	144 ± 5^†^	153 ± 2^†¥^
**MAP (mmHg)**	146 ± 6	164 ± 5^†^	174 ± 4^†¥^
**HR (bpm)**	352 ± 13	348 ± 16	403 ± 12^†¥^
**HRV**			
**SD (ms)**	6.62 ± 0.61	8.63 ± 1.00	5.43 ± 0.96^¥^
**VAR (ms** ^**2**^ **)**	53.97 ± 7.98	63.64 ± 10.34	30.84 ± 6.81^¥^
**LF (ms** ^**2**^ **)**	3.18 ± 0.55	3.94 ± 0.97	0.90 ± 0.12^¥^
**HF (ms** ^**2**^ **)**	5.97 ± 1.41	9.22 ± 2.03	2.77 ± 0.18
**BPV**			
**VAR (mmHg** ^**2**^ **)**	31.08 ± 2.66	51.94 ± 6.94^†^	77.79 ± 11.87^†¥^
**LF (mmHg** ^**2**^ **)**	5.06 ± 0.91	5.07 ± 0.52	10.62 ± 2.33^†¥^

Data are reported as mean ± SEM. ^†^P < 0.05 vs. H; ^¥^P < 0.05 vs. HO. SAP: systolic arterial pressure; DAP: diastolic arterial pressure; MAP: mean arterial pressure; HR: heart rate; Heart rate (HRV) and systolic blood pressure (BPV) variability computed from 0.20 to 3 Hz (total power). SD: standard deviation; VAR: total variance; LF: low-frequency band (0.20-0.75 Hz); HF: high-frequency band (HF: 0.75-3 Hz).

Standard deviation (SD) and total variance VAR of PI were lower in FHO groups when compared to the HO group. Total variance (VAR) of SAP was increased in the HO group when compared to the H group. VAR and LF band of SAP were higher in the FHO group when compared to all other studied groups (Table 2).

**Figure 1:**
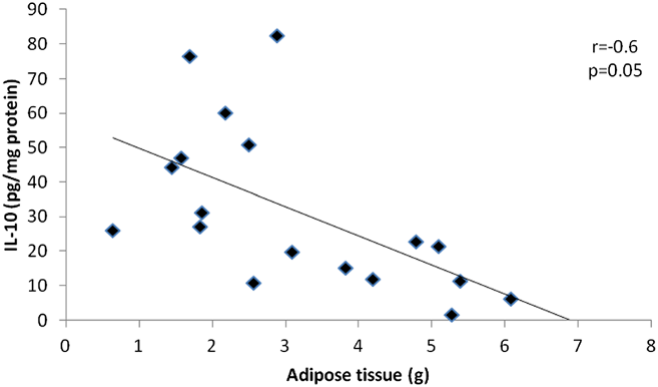
**Negative Pearson correlation (r = -0.6, P = 0.05) obtained between adipose tissue and cardiac levels of IL-10 for studied groups.**

TNFα was higher in the FHO when compared to all other groups and IL-10 was lower in FHO when compared to the H group. Negative correlation was observed between adiposity tissue and IL-10 among hypertensive groups (r = -0.6) (Table 3 and Figure 1).

**Table 3:** **Cardiac inflammatory markers in studied groups**

	H	HO	FHO
**TNF α** (pg/mg protein)	32.9 ± 7.5	31.7 ± 8.6	65.8 ± 9.9^¥†^
**IL-6** (pg/mg protein)	379 ± 27	368 ± 26	401 ± 35
**IL-10** (pg/mg protein)	37.1 ± 9.0	29.6 ± 3.4	16.2 ± 2.5^†^

Data are reported as mean ± SEM. ^†^P < 0.05 vs. H; ^¥^P < 0.05 vs. HO.

TBARS was enhanced in both HO and FHO groups in relation to H group; and additional increase in TBARS was observed in FHO rats when compared to HO rats. Moreover, CL was higher in FHO when compared to all other groups. Hormone deprivation promoted an increase in CAT activity and fructose overload promoted an additional increase in this parameter. We did not observe any difference in SOD activity between groups. GPx was lower in HO group when compared to the H group and fructose overload promoted an additional increase in this parameter (Table 4).

**Table 4:** **Cardiac oxidative stress evaluations in studied groups**

	H	HO	FHO
**TBARS** (μmol/mg protein)	4.95 ± 0.89	8.27 ± 0.98^†^	12.03 ± 0.93^†¥^
**CL** (cps/mg protein)	6661 ± 566	6514 ± 547	15043 ± 1333^†¥^
**CAT** (nmol/mg protein)	0.29 ± 0.04	0.47 ± 0.05^†^	0.63 ± 0.05^†¥^
**SOD** (USOD/mg protein)	11.47 ± 0.37	11.17 ± 0.41	13.05 ± 0.86
**GPx** (nmol/min/mg protein)	54.78 ± 3.10	38.55 ± 1.67^†^	63.39 ± 5.52^¥^
**GSH/GSSG** (nmol/min/mg protein)	11.07 ± 0.7	13.00 ± 1.4	8.94 ± 0.8^¥^

Data are reported as mean ± SEM. ^†^P < 0.05 vs. H; ^¥^P < 0.05 vs. HO.

## Discussion

Gender differences do play a role in the onset of hypertension and other risk factors. The National Health and Nutrition Examination Survey (NHANES) has found that, prior to 45 years of age, prevalence of hypertension is higher in men than in women. From 45 to 54 years and from 55 to 64 years of age, the percentage of hypertensive men is similar to that of hypertensive women, a period which coincides with the advent of menopause. After 65 years of age, BP levels increase faster in women than in men [12]. Moreover, changes in dietary habits are among the leading contributors to the growing worldwide prevalence of metabolic syndrome, obesity, and type 2 diabetes. These changes are characterized by increased intake of simple sugars, particularly fructose, commonly used in the food industry and sugar-sweetened drinks [2]. However, the effects of high calorie intake in hypertensive post menopausal women are not well understood. Therefore, we aimed to assess the effects of fructose overload on cardiovascular autonomic modulation, inflammation and cardiac oxidative stress in an experimental model of association of hypertension and menopause. It should be stressed that fructose overload has been used in animals for inducing insulin resistance, obesity, borderline hypertension, and lipid abnormalities [21]. Furthermore, several studies have used ovariectomy to determine the effects of ovarian hormone deprivation on cardiovascular and metabolic parameters [9, 10]. In the present study, the association of ovarian hormone deprivation in SHR with high intake of fructose led to impairments in cardiovascular autonomic modulation, and worsened both oxidative stress and inflammation parameters.

In the present study, ovarian hormone deprivation increased both AP and vascular sympathetic modulation in SHR rats. Previous studies from our group have also demonstrated that ovarian hormone deprivation induces BP increase and leads to impaired baroreflex sensitivity in healthy Wistar rats [9, 10]. In addition, it should be noted that in the first decades of life, women have a greater cardioprotection when compared to men, as evidenced by increased vagal modulation and lower sympathetic modulation. However, approximately between the fifth and sixth decade of life this greater cardioprotection disappears [7], a period that coincides with the advent of menopause, thus demonstrating the importance of ovarian hormones in cardiovascular control.

Experimentally, fructose overload in drinking water or chow has been used to study metabolic syndrome and promote similar metabolic and autonomic derangements [3, 5, 22, 23]. Indeed, in present study, fructose overload promoted an additional impairment of metabolic, autonomic, oxidative stress and inflammatory parameters in hypertensive ovariectomized rats. In fact, the sum of risk factors (hypertension + ovariectomy + fructose overload) promoted an increase in body weight, adipose tissue and triglyceride concentration along with impaired insulin sensitivity in FHO rats. It has been demonstrated that the exposure of the liver to large quantities of fructose promotes rapid stimulation of lipogenesis, which in turn contributes to triglyceride accumulation, leading to fewer insulin receptors and, consequently, reducing insulin sensitivity [24].

Moreover, the sum of risk factors promoted an increase in the heart rate, an additional increase in BP, impairment in heart rate variability and systolic arterial pressure variability. Several studies have found cardiac diastolic dysfunction and an increase in BP after fructose overload [5, 22, 23, 25]. In the present study, we observed that both DAP and MAP were higher in HO group when compared to the H group and FHO group had an additional increase in SAP, DAP and MAP. One possible explication for this increased BP would be the impairment of cardiovascular autonomic modulation.

Heart rate variability has been used as a tool to assess the role of autonomic nervous system fluctuations in normal healthy individuals and in patients with various cardiovascular and non-cardiovascular disorders [26]. Reduction in HRV has been associated with negative outcomes [27]. In the present study, fructose overload promoted a reduction in HRV as well as an increase in sympathetic activity, as demonstrated by the increase in VAR-SAP and LF-SAP. Moreover, previous studies from our group have shown that fructose overload also promotes an increase in sympathetic tonus, a decrease in vagal tonus and a reduction in baroreflex sensitivity [5].

Regarding inflammation makers, we observed that in this study TNF-α was higher in FHO when compared to other groups while fructose overload promoted a reduction in IL-10. We also found a negative correlation between adipose tissue an IL-10. In fact, adipose tissue is an active endocrine and paracrine organ that releases a large number of cytokines and bioactive mediators, such as leptin, interleukin-6 (IL-6) and tumor necrosis factor-α (TNF-α) [28, 29]. These substances negatively influence glucose and lipid homeostasis, blood pressure, coagulation, fibrinolysis and inflammation, which may lead to endothelial dysfunction and atherosclerosis [28, 29].

Moreover, decreased antioxidant capacity together with increased lipid peroxidation has been reported in patients with fatty liver, visceral obesity, and metabolic syndrome. There seems to be a correlation between the amount of visceral fat and systemic oxidative markers, indicating that visceral fat is an independent regulator of oxidative changes [30]. Regarding antioxidant enzymes, hormone deprivation promoted an increase in CAT activity and a decrease in GPx activity in the HO group when compared to the H group. Ruiz et al. have shown that 17beta-estradiol has intrinsic antioxidant properties, and the lack of it would then result in enhanced CAT activity, probably due to an increased concentration of hydrogen peroxide [31]. Our results are in agreement with Barp et al. [32] who have likewise demonstrated an increased cardiac CAT activity in ovariectomized rats. Moreover, the increase in CAT in the HO group (vs. H) may be also associated with the observed decrease in GPx in this group, since these enzymes compete for the same substrate (hydrogen peroxide). Importantly, lipoperoxidation (TBARS) was increased in SHR ovariectomized rats when compared to intact SHR rats, suggesting increased oxidative stress. A previous study from our group has demonstrated a positive correlation between systolic AP and lipoperoxidation, thus reinforcing the role of oxidative stress in AP changes during female hormone deprivation [9]. However, it should be stressed that, despite favorable changes in antioxidant enzymes in fructose fed group observed in the present work, lipoperoxidation was markedly increased in the fructose group (TBARS and CL) while glutathione redox balance was reduced, thus demonstrating an imbalance between pro-oxidant and antioxidant forces. This would point to increased oxidative stress in rats undergoing association of risk factors.

Regarding the limitations of the present study, we did not evaluate non-OVX groups treated with fructose, which would help us determine whether the combination of OVX and high fructose are additive or synergistic. Furthermore, we used a direct acute measurement of BP, which is a well-established and widely accepted method for BP recording in conscious rats; however, this method have some disadvantages when compared to telemetry AP measurements.

## Conclusions

In conclusion, fructose overload induced an impairment of cardiovascular autonomic modulation associated with increased oxidative stress and inflammatory parameter in hypertensive ovariectomized rats. These data reinforce the role of autonomic changes in immune function modulation [4, 28, 29, 33], by promoting the release of bioactive molecules which are involved in the increased oxidative stress and development of cardiometabolic changes after menopause.
